# The H_3_^+^ ionosphere of Uranus: decades-long cooling and local-time morphology

**DOI:** 10.1098/rsta.2018.0408

**Published:** 2019-08-05

**Authors:** Henrik Melin, L. N. Fletcher, T. S. Stallard, S. Miller, L. M. Trafton, L. Moore, J. O'Donoghue, R. J. Vervack, N. Dello Russo, L. Lamy, C. Tao, M. N. Chowdhury

**Affiliations:** 1 Department of Physics & Astronomy, University of Leicester, Leicester, UK; 2 Department of Physics & Astronomy, University College London, London, UK; 3 Department of Astronomy, University of Texas, Austin, TX, USA; 4 Center for Space Physics, Boston University, Boston, MA, USA; 5 Goddard Space Flight Centre, Greenbelt, MD, USA; 6 Johns Hopkins Applied Physics Laboratory, Laurel, MD, USA; 7 LESIA, Observatoire de Paris, PSL, CNRS, Sorbonne Université, Meudon, France; 8 National Institute of Information and Communications Technology, Tokyo, Japan

**Keywords:** Uranus, aeronomy, spectroscopy

## Abstract

The upper atmosphere of Uranus has been observed to be slowly cooling between 1993 and 2011. New analysis of near-infrared observations of emission from H_3_^+^ obtained between 2012 and 2018 reveals that this cooling trend has continued, showing that the upper atmosphere has cooled for 27 years, longer than the length of a nominal season of 21 years. The new observations have offered greater spatial resolution and higher sensitivity than previous ones, enabling the characterization of the H_3_^+^ intensity as a function of local time. These profiles peak between 13 and 15 h local time, later than models suggest. The NASA Infrared Telescope Facility iSHELL instrument also provides the detection of a bright H_3_^+^ signal on 16 October 2016, rotating into view from the dawn sector. This feature is consistent with an auroral signal, but is the only of its kind present in this comprehensive dataset.

This article is part of a discussion meeting issue ‘Advances in hydrogen molecular ions: H_3_^+^, H_5_^+^ and beyond’.

## 1. Introduction

William Herschel's discovery of Uranus in 1781, announced in this journal [1], marked the first addition to the roster of planets in our Solar System since antiquity. With Herschel's discovery of two moons orbiting the planet 6 years later [2], Uranus was demonstrated to have a very large tilt, with the rotational axis almost aligned with the plane of the ecliptic, producing extreme seasons over its 84 year journey about the Sun. This subjects certain regions of the atmosphere to extreme contrasts, from being fully illuminated by the Sun to being entirely in darkness.

In 1986, the Voyager 2 spacecraft flew past Uranus, providing our first and only detailed close-up view of the planet. It revealed a strange magnetic field, offset approximately 60° from the rotational axis, with the dipole axis offset 0.3 *R*_*U*_ (1 *R*_*U*_ = 25 362 km) from the centre of the planet [3]. With the rotational axis aligned with the ecliptic plane, the magnetic field configuration with respect to the interplanetary magnetic field (IMF) changes dramatically throughout each Uranian day (17.24 ± 0.01 h; [4]), but also throughout the Uranian year (*P* = 84 yr). In addition, the angle of attack between the magnetic dipole and the solar-wind flow varies less at solstice than at equinox. During the flyby, Voyager 2 discovered weak auroral emissions in the ultraviolet, dotted about the magnetic poles [5], with the total emitted energy flux being approximately 50 times weaker than the aurora observed at Saturn (e.g. [5,6]). More recently, observations using the Hubble Space Telescope (HST) [7,8] have re-detected the ultraviolet aurora, imaging the dayside aurora before and after equinox in both hemispheres. Near-equinox aurora are weak, spot-like and intermittent but are observed regularly between 2011 and 2014, with the power, size and occurrence rate all increasing during that time. The HST observations obtained in 2014 were about as bright as those observed by Voyager, and the morphology was described well by model auroral ovals.

Owing to the large offset between the rotational axis and the magnetic dipole axis, auroral emission appears close to the rotational equator, about the magnetic poles, with the HST observations showing emissions at a maximum latitude of −50° [8]. Here, the Uranus Longitude System (ULS) is used [3], which defines the visible pole during the Voyager 2 encounter as the northern, while the International Astronomical Union defines it as the southern. These emissions are likely to be driven by impulsive dayside reconnection, but could also exhibit more stable field-aligned currents that close in the ionosphere. These processes can inject significant amounts of energy into the upper atmosphere by way of Joule heating. Theoretical considerations [9,10] have indicated that the geometry of Uranus at equinox in 2007 was not favourable for building up significant flux in the magnetotail, consistent with weak and intermittent auroral emissions. By contrast, during solstice conditions, reconnection is favourable at the magnetopause and magnetic flux can be accumulated in the tail, conditions favourable for generating aurora. This picture is consistent with a seasonal dependence on the intensity of the observed auroral emissions.

As highlighted throughout this special issue, the molecular ion H_3_^+^ is an important tracer of energy being injected into a system dominated by molecular hydrogen, and by analysing its spectrum one can determine the temperature of the region in which the ions are formed, along with the column integrated line-of-sight density. H_3_^+^ is a very efficient emitter in the infrared and can provide significant radiative cooling in hot environments (e.g. at Jupiter [11]). An increase in the H_3_^+^ temperature can be indicative of localized heating, while the density of H_3_^+^ is determined by the balance between H_2_ ionization rates and the loss via recombination with electrons.

H_3_^+^ emissions from Uranus were discovered on the 1 April 1992 [12] using the United Kingdom Infrared Telescope (UKIRT). In the period between 1993 and 2009, a variety of observing programmes, using a range of ground-based telescopes, recorded H_3_^+^ emissions from Uranus. Melin *et al.* [13] returned to these datasets and self-consistently re-analysed the observations, fitting temperatures and line-of-sight densities to the observed H_3_^+^ spectra. Owing to the relatively low signal to noise (S/N) of the data and small angular size of Uranus, these temperatures represent globally averaged temperatures, providing a unique view of the ionosphere as a unitary system. In this time series, they discovered that the upper atmosphere of Uranus had dramatically cooled from 715 ± 47 K in 1992 to 534 ± 39 K in 2008. This slow yet consistent cooling was initially interpreted to be related to seasonal solar irradiance, modulating the effectiveness of the Joule heating by changing ionospheric conductivity, where the solstice (1986) would appear hotter than the equinox (2007) since the illuminated area on the planet over the course of a day is greater by about a factor 2 at equinox. This hypothesis predicted that the cooling of the upper atmosphere would reverse to an epoch of heating at equinox, after some time-lag governed by the thermal insulation of this region. However, subsequent observations in 2011 [14] revealed that the upper atmosphere had continued to cool further, down to 520 ± 32 K, indicating either that the thermal lag is extremely long, or that the hypothesis of purely solar-driven seasons in the upper atmosphere is incomplete.

On a much larger perspective, one of the outstanding questions in giant planet aeronomy is why the upper atmosphere of all these planets is several hundreds of degrees Kelvin hotter than solar input alone can produce. Both heating by global re-distribution of auroral energy injected about the magnetic poles, and heating by breaking of acoustic or gravity waves generated by the turbulent lower atmosphere have been proposed as potential solutions to this ‘energy crisis’. There is evidence that both of these could contribute significant heat under specific circumstances (for Jupiter, see e.g. [15,16]), but a cohesive picture that completes the energy budget at all the giant planets is still missing. Understanding how the temperature of Uranus' upper atmosphere evolves over time will add important constraints to our understanding of these processes.

This study extends the baseline of observations with ones obtained in 2012– 2018, bringing the total period of H_3_^+^ observations of Uranus to 27 years, longer than an individual solar season at Uranus (21 years), adding data from the NASA Infrared Telescope Facility (IRTF), Keck, the Very Large Telescope (VLT) and Gemini. These facilities also enable the characterization of the H_3_^+^ emission across the disc of Uranus.

## 2. Observations

Because of the small angular size of Uranus in the sky (approx. 3.7^′′^) and large distance from the Sun (approx. 19 AU), the H_3_^+^ signal observed from the planet generally has low S/N. This, in combination with the large pixel scales of early instrumentation (e.g. [12] used a 3.1^′′^/pixel instrument) means that the temperatures derived by Melin *et al.* [13,14] are effectively a global average over the period of observation, which is often a significant fraction of complete longitude coverage. More recent observations with smaller pixel scales, narrower slits and higher sensitivity, allow a more detailed view of the ionosphere of Uranus. However, in order to be able to compare like-for-like with historical observations, the more recent observations have initially been averaged over each night of observation to produce a similar global view of the ionosphere for each particular epoch. This works particularly well when the spectrograph slit is scanned across the disc of the planet, providing broader latitude coverage. Throughout this paper, the ULS [3] is employed, defining the north pole as the sunlit pole during the Voyager 2 encounter.

Here we analyse H_3_^+^ observations of Uranus obtained between 2012 and 2018. This includes observations from five different instruments on four different telescopes. Table 1 details the observations analysed in this study, listing the observations day-of-year (DOY) and mid-time (UTC), the length of time between the first and the last exposure, the calibration star used (all of spectral type A0), and the telescope-specific programme ID. Figure 2 shows an example spectrum from each instrument used in this study, and a brief description of each is provided below.

**Table 1.** The separate nights of mid-infrared observations of H_3_^+^ emission from Uranus analysed in this study. The mid-observation day-of-year (DOY) and UTC time are listed, along with the time between the first and the last exposure, the A0 calibration star used and the telescope specific programme ID.

ID	facility and instrument	DOY mid-obs	*t* (h)	Cal. Star	program ID
1	NASA IRTF SpeX	2012-229 13.15	4.8	HR 8911	2012B070
2	NASA IRTF SpeX	2012-321 07.04	4.6	HR 8911	2012B070
3	Gemini GNIRS	2012-348 06.36	3.2	HR 718	GN-2012B-Q-114
4	NASA IRTF SpeX	2013-215 13.12	4.6	HR 8573	2013B010
5	NASA IRTF SpeX	2013-216 13.24	4.2	HR 8573	2013B010
6	VLT CRIRES	2013-295 04.44	3.2	HR 8911	092.C-0077(A)
7	VLT CRIRES	2013-296 04.54	4.6	HR 8911	092.C-0077(A)
8	VLT CRIRES	2013-297 02.54	1.9	HR 125	092.C-0077(A)
9	VLT CRIRES	2013-300 04.08	3.8	HR 125	092.C-0077(A)
10	NASA IRTF SpeX	2013-319 09.32	4.2	HR 658	2013B010
11	Gemini North GNIRS	2013-340 08.08	2.5	HR 378	GN-2013B-Q-93
12	Gemini North GNIRS	2013-359 06.14	2.2	HR 8826	GN-2013B-Q-93
13	NASA IRTF SpeX	2014-255 12.04	5.2	HR 658	2014B034
14	NASA IRTF SpeX	2014-256 12.22	4.6	HR 658	2014B034
15	Keck II NIRSPEC	2014-285 11.29	0.9	HR 718	2014BN122NS
16	Keck II NIRSPEC	2014-286 11.15	1.3	HR 718	2014BN122NS
17	NASA IRTF SpeX	2014-328 06.28	3.0	HR 8518	2014B034
18	NASA IRTF SpeX	2014-337 07.26	5.2	HR 8518	2014B034
19	NASA IRTF SpeX	2015-325 08.16	7.1	HR 658	2015B031
20	NASA IRTF iSHELL	2016-284 09.32	4.1	HR 311	2016A041
21	NASA IRTF iSHELL	2016-285 10.15	5.5	HR 8518	2016A041
22	NASA IRTF iSHELL	2016-320 07.42	6.0	HR 8518	2016A041
23	NASA IRTF iSHELL	2017-230 14.58	1.2	HR 840	2017B077^a^
24	NASA IRTF iSHELL	2017-244 13.30	1.2	HR 8865	2017A037
25	NASA IRTF iSHELL	2017-245 09.52	1.3	HR 8865	2017A037
26	NASA IRTF iSHELL	2017-258 12.56	5.1	HR 1718	2017A037
27	NASA IRTF iSHELL	2017-259 12.58	5.2	HR 1718	2017A037
28	NASA IRTF iSHELL	2017-260 12.56	5.1	HR 1718	2017A037
29	NASA IRTF iSHELL	2018-287 12.53	4.1	HR 1061	2018A037
30	NASA IRTF iSHELL	2018-288 11.33	6.8	HR 1061	2018A037
31	NASA IRTF iSHELL	2018-312 09.36	6.7	HR 7981	2018A037
32	NASA IRTF iSHELL	2018-314 08.54	8.1	HR 7891	2018A037

^a^This dataset was published as reference spectra in the search for H_3_^+^ at Neptune [17].

### (a) NASA IRTF iSHELL

The NASA IRTF iSHELL instrument [18] is a high-resolution spectrograph that became operational in August 2016, with Uranus being the first science target after commissioning (programme 2016A041). Using the *Lp*3 setting and the 0.375^′′^ × 15^′′^ slit produces a cross dispersed spectra on a 2048 × 2048 pixel detector, at a spectral resolution of *R*∼70 000. The spectral range is 3.83–4.14 μm, giving a near complete coverage of the H_3_^+^ *Q* branch about 4 μm. Once extracted, a complete spectrum contains over 28 000 spectral pixels.

During these observations, the spectrograph slit was scanned across the disc of the planet, using three spatial positions, each separated by 0.8^′′^, centred about the centre of the disc. An example iSHELL spectrum can be seen in figure 2*a*, with the dashed lines indicating regions where the spectra have been truncated in order to emphasize the individual H_3_^+^ lines.

### (b) Keck NIRSPEC

The Near-Infrared Spectrograph (NIRSPEC; [19]) is a medium- to high-resolution spectrograph mounted on the Keck II telescope on the summit of Mauna Kea in Hawaii, USA. Using an echelle angle of 62.02° and a cross disperser angle of 33.65°, with the 0.432^′′^ × 24^′′^ slit, produces a cross dispersed spectrum covering the H_3_^+^ *Q* branch region about 4 μm at a spectral resolution of *R*∼25 000. A NIRSPEC H_3_^+^ spectrum is shown in figure 2*b*.

### (c) Gemini GNIRS

The Gemini Near InfraRed Spectrograph (GNIRS; [20]) is a long-slit medium resolution spectrograph mounted on Gemini North on Mauna Kea, Hawaii. Using the 32 lines mm^−1^ grating and the 0.1^′′^ wide slit in the *L* band produces a spectrum with a spectral resolution of *R*∼5400. These observations were designed to be acquired during ‘Band 3’ conditions, which includes periods of significant cloud-cover and high water vapour content in Earth's atmosphere, unsuitable for the majority of science programmes. An example Gemini GNIRS spectrum is shown in figure 2*c*.

### (d) NASA IRTF SpeX

**Figure 1.**
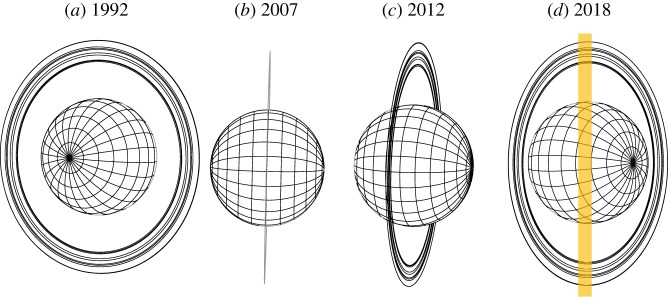
The geometry of Uranus as seen from Earth in 1992, 2007, 2012 and 2018. The last uranian equinox was in 2007 and the next solstice will be in 2028. The rings are not visible at L^′^ (approx. 4.0 μm) in the near-infrared. The orientation of the spectrograph slit is shown in orange for 2018, with the same orientation used for all the data used in this study.

**Figure 2.**
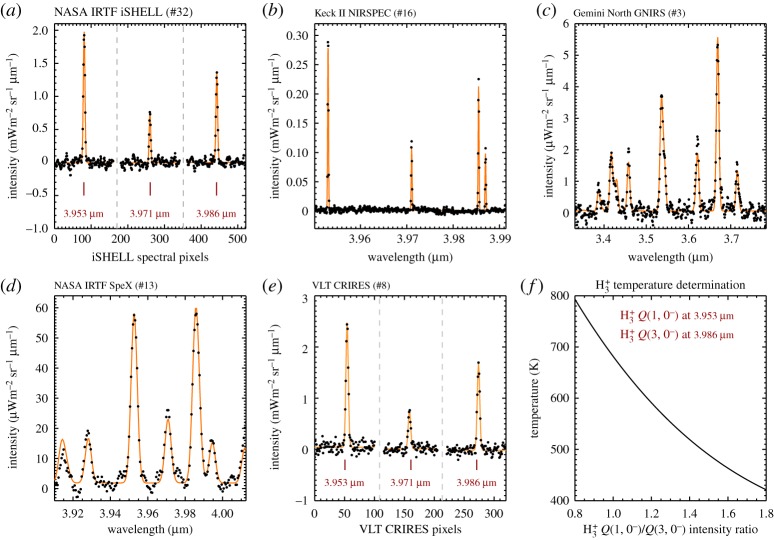
(*a*–*e*) Example Uranus H_3_^+^ spectra, one from each of the facilities and instruments used in this study. The very high-resolution spectra shown in (*a*) and (*e*) show the spectral regions containing three H_3_^+^ *Q* branch transitions as a function of spectral pixel due to the high spectral dispersion, where the dashed lines indicate a discontinuity in the wavelength coverage. (*f* ) The dependence of the H_3_^+^ *Q*(1, 0^−^)/*Q*(3, 0^−^) ratio on temperature.

The NASA IRTF SpeX instrument [21] is a medium resolution spectrograph, here operated at the setting *LongXD1.9* giving a spectral resolution of *R*∼2500 with the 0.5^′′^ × 15^′′^ slit. The wavelength coverage encompasses the H_3_^+^ *Q* branch spectrum. The instrument was upgraded to a Teledyne H2RG detector array in August 2014, increasing the sensitivity and pixel density. These observations employed a single slit-position across the centre of the disc of Uranus, as indicated in figure 1*b*. An example SpeX H_3_^+^ spectrum is shown in figure 2*d*.

### (e) VLT CRIRES

The CRIRES instrument [22] mounted on the VLT Unit Telescope 3 (UT3) is a long-slit spectrograph, capable of very high spectral resolution. The 0.2^′′^ × 25^′′^ slit and a central wavelength of 3.965 μm produces a single-order spectrum distributed across four Raytheon detectors, at a resolution of *R*∼100 000, covering the *Q* branch of H_3_^+^. CRIRES was removed from UT3 in mid-2014 for a component upgrade, due to return to the telescope in late 2019. The high-resolution H_3_^+^ spectrum observed by CRIRES is shown in figure 2*e*, with the dashed lines separating the three very narrow spectral regions.

## 3. Analysis

### (a) Long-term evolution of the temperature of the ionosphere

The first-order data product obtained from each night of observations is a spectrum of intensity versus wavelength, obtained in the *L* telluric window, between 3.3 and 4.1 μm (e.g. figure 2). At Uranus, this wavelength region contains almost exclusively emission from H_3_^+^, since methane in the stratosphere absorbs any incident sunlight or thermal emission generated below the homopause. (This is in contrast to Jupiter and, particularly, Saturn.) This greatly simplifies the analysis of the observed spectrum. The *L* band window contains emission principally from the *R* and *Q* branch of H_3_^+^, regions with a rich history of being used as a probe for determining the temperatures of the upper atmospheres of the giant planets. In particular, the intensity ratio between the H_3_^+^ *Q*(1, 0^−^) at 3.953 μm and the *Q*(3, 0^−^) at 3.986 μm lines provides an excellent measure of temperature in the range of 400–800 K, as illustrated in figure 2*f* . This line-ratio is used to derive the temperature for the VLT CRIRES and NASA IRTF iSHELL observations in table 1, since the spectral resolution is high enough to resolve individual spectral lines. For the rest of the data, at lower spectral resolution, a full line-by-line H_3_^+^ model of the appropriate spectral region containing multiple lines is fitted to a temperature by applying Cramer's rule (e.g. [14,23]). There is virtually no difference in the retrieved temperature between the two methods. The H_3_^+^ transitions are modelled using the line list of Neale *et al.* [24] and the partition function of Miller *et al.* [25], assuming conditions of *q*-LTE [24]. The error on the retrieved parameters is principally governed by the S/N (for more details, see [23]).

Once the temperature is determined from the shape of the H_3_^+^ spectrum, the number of emitting molecules is calculated by dividing the observed intensity of the *Q*(1, 0^−^) line by the emission per molecule at the derived temperature. The total emission, which is the energy radiated over all wavelengths, is then calculated using the formulation of Miller *et al.* [26].

**Figure 3.**
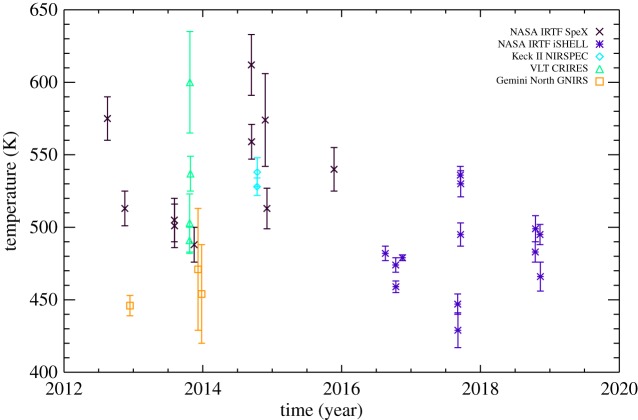
The H_3_^+^ temperatures derived for individual observing nights listed in table 1. There is significant variability in temperature between each individual observation. The maximum measured temperature is 612 K in 2014 and the minimum is 429 K in 2017 with a mean of 507 K. The standard deviation is 44 K.

Table 2 shows the retrieved H_3_^+^ temperatures, H_3_^+^ densities, with associated errors, for each night of observation listed in table 1. For completeness, it also lists the calculated H_3_^+^ total emission, which can be an effective cooling mechanism for the upper atmosphere [25]. The temperature for each observing night is plotted in figure 3. The maximum measured temperature is 612 K in 2014 and the minimum is 429 K in 2017 with a mean of 507 K. The standard deviation is 44 K.

**Table 2.** The retrieved global H_3_^+^ temperature (*T*), temperature error (Δ*T*), column density (*N*, in units of 10^15^ m^−2^), column density error (Δ*N*) and the wavelength integrated H_3_^+^ emission (*E*, in units of μWm^−2^ sr^−1^).

ID	DOY	*T* (K)	Δ*T*	*N*^1^	Δ*N*	*E*
1	2012-229	575	15	2.2	0.4	3.9
2	2012-321	519	12	11.6	2.2	10.3
3	2012-348	446	7	1.7	0.2	0.5
4	2013-215	505	15	9.7	2.3	7.1
5	2013-216	501	15	6.4	1.5	4.5
6	2013-295	491	9	3.0	0.4	1.8
7	2013-296	503	20	3.8	1.3	2.7
8	2013-297	600	35	2.1	0.6	4.9
9	2013-300	537	12	12	2	10.1
10	2013-319	486	12	14.3	3.0	7.9
11	2013-340	471	42	8.7	5.7	3.8
12	2013-359	454	34	6.0	3.5	1.9
13	2014-255	612	21	1.1	0.3	3.0
14	2014-256	559	12	2.5	0.4	3.7
15	2014-285	528	6	1.0	0.1	1.0
16	2014-286	538	10	7.9	1.1	9.0
17	2014-328	574	32	3.3	1.3	5.9
18	2014-337	513	14	3.5	0.8	2.9
19	2015-325	540	15	1.8	0.4	2.1
20	2016-230	482	5	6.8	2.0	3.5
21	2016-284	474	5	6.3	1.9	2.9
22	2016-285	459	4	8.6	2.6	3.1
23	2016-320	479	2	6.2	1.9	3.1
24	2017-244	447	7	9.3	2.8	2.7
25	2017-245	429	12	5.0	1.5	1.0
26	2017-258	536	6	1.5	0.4	1.7
27	2017-259	495	8	2.0	0.6	1.3
28	2017-260	530	9	1.7	0.5	1.8
29	2018-287	483	7	4.2	1.3	2.2
30	2018-288	499	9	4.6	1.4	3.1
31	2018-312	495	7	2.2	0.6	1.4
32	2018-314	466	10	2.7	0.8	1.1

**Figure 4.**
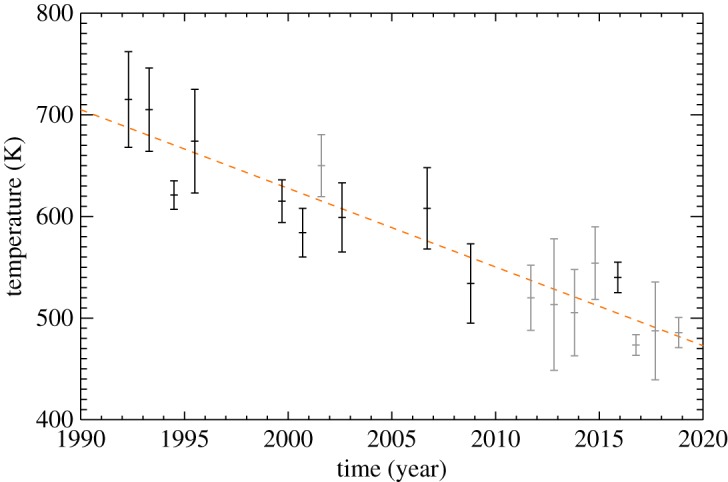
The yearly average temperature of the upper atmosphere of Uranus as derived from the H_3_^+^ observations, between 1992 and 2018, showing a long-term cooling trend. The dashed line is a linear fit to the data, with a slope of 8 ± 1 K yr^−1^. The plotted temperatures are listed in table 3. The grey error bars indicate years with multiple observations, with black errors bars indicating single measurements.

There is significant variability in the temperatures in figure 3. In order to investigate how these observations fit in with the long-term observations [13,14], a yearly temperature average is calculated from the observations contained in table 2. These averages are listed in table 3 and are shown together with the yearly averaged temperatures of previous studies in figure 4. The error bars represent the standard deviation of the observations within a particular year (grey), unless there is only one data-point, in which case the error bar is the uncertainty on the temperature (black). A linear fit to the globally averaged temperature between 1992 and 2018 shows that the ionosphere has continued to cool at a steady rate of 8 ± 1 K yr^−1^—the same rate as derived by the initial long-term analysis [13].

**Table 3.** The yearly averaged global parameters, calculated from table 2, listing H_3_^+^ temperature (*T*), standard deviation on the temperature (*σT*), column density (*N*, in units of 10^15^ m^−2^), the standard deviation on the column densities (*σN*) and the wavelength integrated H_3_^+^ emission (*E*, in units of μWm^−2^ sr^−1^).

year	*T* (K)	*σ*(*T*)	*N*	*σ*(*N*)	*E*
1992.3	715	47	1.4	0.3	9.0
1993.3	705	41	1.4	0.4	8.4
1994.5	621	14	2.9	0.2	8.3
1995.5	674	51	1.2	0.4	5.6
1999.7	615	21	3.9	1.6	10.6
2000.7	584	24	3.4	0.0	6.7
2001.6	650	31	1.8	0.5	6.6
2002.6	599	34	1.9	0.5	4.4
2006.7	608	40	1.4	0.8	3.5
2008.8	534	39	1.6	0.5	1.7
2011.7	520	32	3.6	0.3	3.2
2012.8	513	65	5.2	0.6	4.2
2013.8	505	42	7.0	5.6	5.1
2014.8	554	36	3.2	3.9	4.5
2015.9	540	15	1.8	2.5	2.1
2016.8	474	10	7.0	0.4	3.1
2017.7	487	48	3.9	1.1	2.2
2018.8	486	15	3.4	3.3	1.9

### (b) Local-time profiles of the ionosphere

With new instruments and telescopes providing greater sensitivity and finer spatial resolution, we are able to move from investigating global properties of the ionosphere to spatially resolving the disc of Uranus. Another important difference between the observations obtained for this study and previous studies [13,14] is that the slit was aligned with the equator, as illustrated in figure 1*d*, as opposed to along the rotational axis. This provides a local-time view of the H_3_^+^ ionosphere, from dusk, across noon, to dawn. Note that these profiles cut across a range of latitudes and longitudes as illustrated in figure 1*b*.

Close to dawn and dusk, the line-of-sight vector traverses a long pathway in the ionosphere, due to the slant viewing angle. This has the effect of increasing the intensity of the observed H_3_^+^ emission. In order to convert these to represent the intensity observed along the ‘surface’ normal, a line-of-sight correction is applied (e.g. [11]).

**Figure 5.**
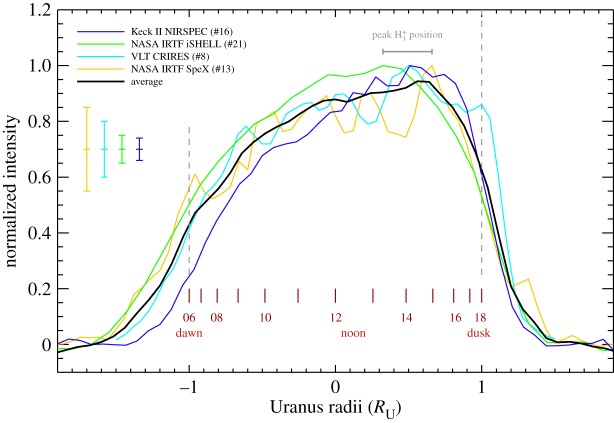
Four local-time H_3_^+^ profiles of Uranus as a function of planetary radius, with the ID of the observation used is indicated. For each observations, the slit was aligned with the equator of the planet. The red lines show the local time across the disk of the planet. The profiles are broadly consistent, peaking in the afternoon sector, between 13.00 and 15.00. The small-scale variability across the disk of Uranus are indicative of the S/N, with uncertainties indicated by the error bars on the left, and are not to be considered real.

Figure 5 shows the line-of-sight corrected local-time H_3_^+^ *Q*(1, 0^−^) intensity profile for four different instruments, as indicated by the observation ID (table 1). The four different instruments produce profiles with vastly different S/N, with NASA IRTF SpeX providing the lowest, and Keck NIRSPEC the highest; the small-scale variability across the disc should not be considered to be real, and instead just indicative of S/N. For the four observations shown in figure 5 the H_3_^+^ intensity peaks between a local time of about 13 and 15. The morning sector is consistently less intense than the evening sector, and the fall-off of intensity outside the ± 1 planetary radius is caused by the telluric seeing.

### (c) The event of 2016-285

The typical ionospheric local-time profile described in the previous sections are present in the observations with good telluric seeing conditions and stable telescope guiding. However, there is one outlier to this relatively stable ionospheric profile: the NASA IRTF iSHELL observations of 2016-285 (#21 in table 1).

**Figure 6.**
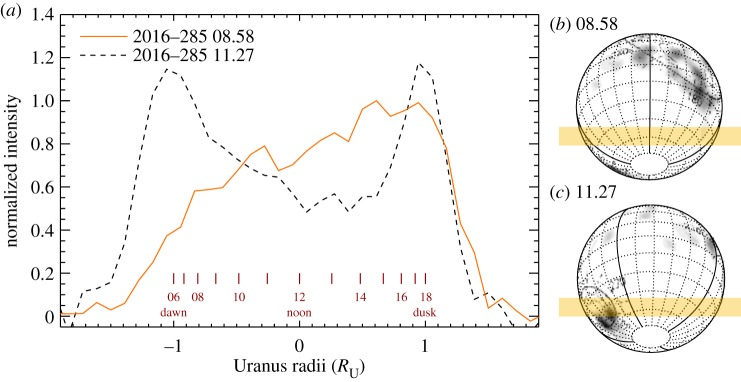
(*a*) Two local-time profiles from the NASA IRTF iSHELL observations obtained on 2016-285 (#22 in table 1), separated by 2.5 h. A clear intensity feature rotates into view. Note that these profiles have not been line-of-sight corrected, effectively enhancing the limb emissions. (*b*,*c*) The geometry of Uranus on 2016-285, with the orientation of the slit shaded in orange. The Voyager 2 auroral observations of Herbert [5] are mapped onto the planet, using a longitude shift consistent with the observations in (*a*).

Figure 6*a* shows two local-time profiles of H_3_^+^ intensity obtained on 2016-285 (#21), with the slit offset 0.8^′′^ south from the centre of the disc, as illustrated in figure 6*b*,*c*. The two observations are separated by 2.5 h, and the profiles have not been line-of-sight corrected to better reveal emissions close to the limb. The profile obtained at 08.58 UTC is similar to the profiles shown in figure 5, increasing towards the dusk sector. The second dashed profile, with a mid-observation time of 11.27 UTC, shows a strong enhancement in the observed H_3_^+^ intensity on the dawn side. Each profile has an integration time of 30 min.

The dashed profile in figure 6*a* appears to be subject to stronger line-of-sight enhancement, which could be caused by a decrease in the telluric seeing, effectively producing a sharper intensity along the sit. The dashed line also appears less intense than the earlier solid intensity profile at local times between 12 and 16 h. This could in part be due to the different seeing conditions experienced during the two observations, but could also be indicative of a H_3_^+^ intensity feature fixed in local time rotating from the afternoon sector (solid line) to the dusk limb (dashed line), producing an increase in the line-of-sight brightening.

The dawn feature seen in the dashed line is consistent with a region of bright H_3_^+^ emission rotating in from the night-side, appearing on the limb, being subjected to strong line-of-sight brightening. At 2016-285 11.27, the sub-observer ULS longitude is 352^°′^ which means that a longitude on the dawn limb is 82°. The exact location of the auroral emission is subject to errors governed by the spatial resolution (approx. 0.7^′′^), total integration time of the co-added observations, and the unknown spatial extension of the feature, placing the emission at a longitude of 82° ± 30°. The latitude of the H_3_^+^ intensity enhancement is −45° ± 15°, consistent with the southern auroral region [8].

Unfortunately, due to observing time constraints, the potential auroral feature in figure 6 could not be tracked across the disc of Uranus, and the observation on 2016-285 remains the only observation with a possible auroral signal present in the entire dataset listed in table 1.

## 4. Discussion

The analysis of the observations of H_3_^+^ outlined above have revealed three important features of the upper atmosphere of Uranus. Firstly, the upper atmosphere has continued to cool since H_3_^+^ was discovered at Uranus in 1992 [12]. Secondly, spatially resolved observations reveal that the peak H_3_^+^ intensity occurs between 13 and 15 in local time. Thirdly, on 2016-285 a dawn brightening appeared in the H_3_^+^ intensity, potentially providing the first detection of H_3_^+^ auroral emission. These results are discussed in greater detail below.

### (a) Long-term cooling in the upper atmosphere of Uranus

The long-term cooling of the upper atmosphere of Uranus discovered by Melin *et al.* [13] has continued throughout the new set of observations analysed in this study. Figure 4 shows a remarkable persistent cooling, from 1992 to 2018, with a linear gradient of 8 ± 1 K yr^−1^. This 27 year interval is longer than the length of a nominal season at Uranus ($\frac{84}{4}=21$ yr), supporting the idea that the heating mechanism, and ultimately the solution to the ‘energy crisis’, is not in any way related to the deposition of solar photons. In addition, neither the passing of equinox (2007) nor aphelion (2009) had any effect on the observed temperatures.

**Figure 7.**
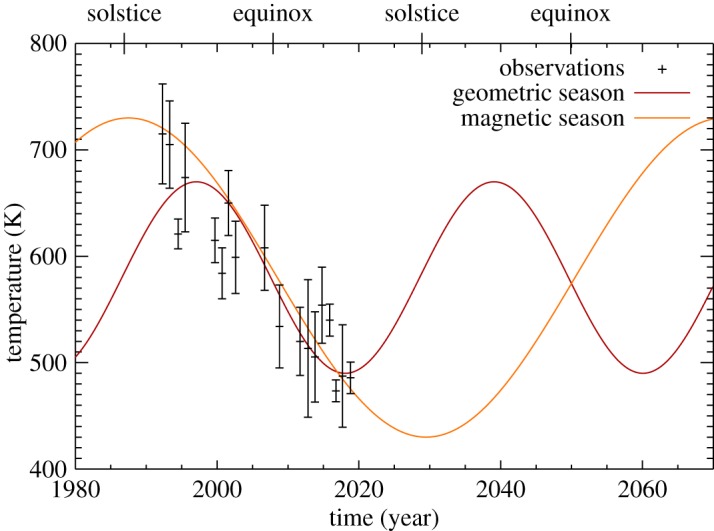
The long-term temperature trend compared to the geometric and magnetic season described in the text.

Owing to the extreme seasons of Uranus, at solstice one pole is in permanent sunlight, while the other is in complete darkness. If the temperature of the ionosphere is governed principally by solar irradiation, then the planet, as observed from Earth, would be subject to two hot solstices and two cool equinoxes. This ‘geometric season’ is illustrated as the red line in figure 7.

Long-term changes in the reflectivity of the troposphere [27] have been linked to the extreme seasons that the planet experiences during its 84 year orbit around the Sun, modulated by the 11 year solar cycle. This suggests that the changing levels of ultraviolet irradiation, or changing rates of solar-modulated galactic cosmic rays, change the photochemistry in the atmosphere. In principle, a similar modulation should be observed in the ionosphere, as the changing solar extreme ultraviolet (EUV) flux throughout the solar cycle would give rise to changing levels of ionization of molecular hydrogen, producing different amounts of H_3_^+^. However, there is no clear correlation between solar cycle and the observed H_3_^+^ column density in the data analysed here (as also noted by Melin *et al*. [13]).

The potential inability for Uranus to build up significant magnetic flux in the tail during equinox conditions compared to solstice [9] provides a mechanism with which the auroral process is modulated by season. Since the magnetic field is both tilted and offset from the centre of the planet, the two solstices will not have the same magnetic configuration with respect to the IMF. This in turn may produce conditions favourable for sustaining currents that drive Joule heating at one solstice, but perhaps not the other, producing one hot and one cold solstice. This ‘magnetic season’ is plotted as the orange line in figure 7. Disentangling these two hypotheses, geometric or magnetic season, requires ongoing observations of the temperature of Uranus' upper atmosphere. Note that both the amplitude and the phase of the solid lines in figure 7 are determined by eye to provide an approximate fit to the data, and serves to illustrate the concept of ‘geometric’ and ‘magnetic’ season.

While the tropospheric reflectance changes as a function of solar cycle, there is no evidence for any long-term changes in the tropospheric temperature [28–30], suggesting that despite being subject to extreme seasons, the thermal effect on the troposphere, as observed from the Earth, is negligible. This is in stark contrast to the long-term cooling observed in the upper atmosphere.

The short-term variability in the temperatures in figure 3 is of the order of approximately 100 K over days. Since the heat capacity of the upper atmosphere of Uranus is likely very large (15 000 Jkg^−1^ K^−1^ for Saturn, [31]), it is unlikely that the variability is due to actual bulk temperature changes. Instead, we expect any short-term changes in temperature to be driven by differences in the peak altitude of H_3_^+^ production, sampling different regions of the thermospheric temperature curve, where low altitudes correspond to lower temperatures, and vice versa [32]. This means that H_3_^+^ is probably predominantly produced along the steep slope of the temperature profile at lower altitudes, and that relatively modest changes in the ionizing energy can produce large differences in the observed H_3_^+^ temperature. In figure 3, there are 2 years where the temperature variability is small, 2016 and 2018, suggesting that the ionization energy remained relatively constant, which may be related to this being the descending phase of solar cycle. Overall, however, given the large numbers of observations used for this study, we expect the yearly averaged temperatures to be representative of the average thermospheric temperature at a fixed altitude, such that the long-term cooling is physical.

### (b) Possible effect of tropospheric storms on the upper atmosphere

Most of the temperatures observed in 2014 are hotter than those observed in 2013, with an average of 505 ± 42 K in 2013 and 554 ± 36 K in 2014. This may be indicative of an intermittent heating event. The uptick of the average global temperature in 2014 is distinct, and coincides with the outbreak of a tropospheric storm in August 2014, fierce enough to punch through the stratospheric methane haze layer [33]. Analogous to the heating that was observed above the Great Red Spot (GRS) of Jupiter [15], this turbulent storm could generate acoustic or gravity waves that traverse vertically up through the atmosphere, breaking and depositing energy in the upper atmosphere, heating this region in the process. At Jupiter, however, the observed heating was highly localized in both longitude and latitude, confined to the region immediately above the GRS. The observations of Uranus presented here cannot spatially resolve the precise location of the heating.

There are three ways in which a tropospheric storm protruding into the stratosphere could produce an apparent heating in the upper atmosphere. Firstly, the storm could generate acoustic or gravity waves that propagate up into the upper atmosphere where they break and deposit their energy. Secondly, an upwelling of stratospheric hydrocarbons into the upper atmosphere provides an efficient sink for H_3_^+^ ions, since hydrocarbons quickly destroy H_3_^+^. Because this would predominantly affect regions close to the homopause, where the temperature is relatively low, an observation of the entire column of H_3_^+^ would therefore exclude the cold population at low altitudes, and therefore result in a higher observed temperature. Thirdly, the breaking of waves generated in the lower atmosphere induce a vertical wind shear in the thermosphere, producing a jagged appearance of the electron density altitude profile, in which case the effective H_3_^+^ peak can be shifted to higher altitudes [34], where it is hotter, producing a higher observed H_3_^+^ temperature.

Heating by tropospheric storms may explain the fact that the temperature is hotter in 2014 than in 2013, while the column density is lower. If the explanation of advecting methane into the upper atmosphere is valid, the particularly low density of H_3_^+^ noted in 2015 might suggest that this methane remained, or was replenished, in the upper atmosphere for at least a year.

### (c) Local-time profiles of the ionosphere

Figure 5 shows four typical local-time profiles contained within the dataset analysed here, with the peak intensity occurring at local times between 13 and 15. The peak ionization rate is expected to occur at local noon, and smaller solar-zenith angles produce higher ionization rates. Unless the temperature changes dramatically across local times, then the location of the observed intensity maximum is also the location of the peak column integrated H_3_^+^ density. It is currently unclear how to shift the peak density away from noon, where the production of H_3_^+^ peaks, but the results obtained here do serve as important input to models of Uranus' upper atmosphere.

Trafton *et al.* [35] observed the intensity of H_3_^+^ across the disc of the planet with the slit aligned along the rotational axis, along local noon, with the maximum intensity occurring at the centre of the disc. By contrast, they also observed the quadrupole H_2_ emission in the *K* telluric window at 2 μm, which appeared to have a line-of-sight enhanced signal, being bright at both limbs. Here, while using a slit aligned along the equator of Uranus, the H_3_^+^ is observed to be subject to strong line-of-sight effects (e.g. figure 6) in a similar manner to the H_2_ emissions.

Imaging performed with the Gemini telescope in 2017 using a Br-*α* filter (4.05–4.10 μm) showed the H_3_^+^ intensity across the peak had a spatial distribution similar to the stratospheric haze [36], with a bright cap above the southern polar region [37], with local-time profiles similar to the ones shown in figure 5. The upwelling of stratospheric hydrocarbons at low latitudes and downwelling at high latitudes [38] could in principle remove a sink of H_3_^+^ at high latitudes, generating brighter emission due to an enhanced H_3_^+^ density. However, this should result in higher H_3_^+^ column densities about solstice, since polar latitudes are predominantly visible from the Earth. There is no seasonal variability, or any clear long-term trends seen the H_3_^+^ densities listed in table 2.

### (d) The dawn H_3_^+^ feature on 2016-285

Figure 6*a* shows a bright H_3_^+^ intensity feature rotate into view on the dawn terminator of the planet. The intensity of H_3_^+^ is driven linearly by density and exponentially by temperature, so an intensity enhancement requires either an increased ionization of H_2_, which very quickly produces H_3_^+^, or a localized increase in temperature. Ionization by solar EUV photons has a well-defined local-time profile, shown in figure 5, and cannot produce a strong enhancement at dawn. The auroral process, however, drives both ionization and heating in the upper atmosphere, and can produce auroral signatures about the magnetic poles throughout all local times [5,8], including the night-side.

The HST observations of Lamy *et al.* [7,8,36] show that the total radiated power of Uranus' aurora increases over time between 2011 and 2017, suggesting an increase in the electron energy flux. The simultaneous HST and Gemini H_3_^+^ imaging observations obtained in 2017 [36] showed that while an auroral feature was clearly seen in the ultraviolet, a corresponding brightening was not observed in H_3_^+^. Since H_3_^+^ emission is strongly dependent on the temperature of the upper atmosphere, any density enhancements of H_3_^+^ generated by the auroral process will have a lower contrast when the upper atmosphere is cold (e.g. 2017), compared to when it is observed to be hot (e.g. 1992)

The ULS longitude of the auroral feature is 82° ± 30°. This location is broadly consistent with the HST observations of auroral emission in the ultraviolet obtained in 2012 by Lamy *et al.* [7], but are different by about 150° longitude with the 2014 observations [8]. However, the rotation period of Uranus has a relatively large uncertainty, which means that over the course of a year an uncertainty of 106° has accumulated, and the absolute longitude is effectively lost. Future spatially resolved, high sensitivity observations of H_3_^+^ auroral observations can be used to re-establish the ULS.

The solar wind propagation model of Tao *et al.* [39] predicts a spike in the solar wind dynamic pressure of approximately 0.03 nPa on 2016-292, 7 days after our observation. The magnitude of this compression is similar to that reported by Lamy *et al.* [7]. At 19 AU, the model is subject to large errors in the arrival time of these enhancements, about ± 3 days, so the observed H_3_^+^ brightening is unlikely to be linked to a solar wind compression. Overall, over 60 h of NASA IRTF iSHELL data was obtained for this study, with only about 2 h showing this clear dawn enhancement, yielding a probability of observing such an event approximately 3%, rendering it very rare indeed.

The fidelity of the data does not allow for an accurate determination of temperature of the dawn feature, but if the dawn H_3_^+^ enhancement is driven by temperature, then this ‘hotspot’ could be produced by a storm in the lower atmosphere, dumping heat into the upper atmosphere. However, no contemporaneous reports of a tropospheric storm have been communicated.

The apparent depletion of H_3_^+^ intensity at noon of the dashed profile in figure 6 may also be interpreted as a region of low intensity H_3_^+^ rotating from dawn in the dashed profile to about noon in the dashed profile. This can either be in the form of a cold region fixed in local time or a density depletion.

More broadly, the short-term variability in both H_3_^+^ column density and temperature could be indicative of auroral features, which are almost always present and varying, but cannot be resolved due to the limited spatial resolution of most of the observations in table 1. The early observations of Lam *et al.* [40] detected variations in the H_3_^+^ intensity of about 20%, while the temperatures in table 2 have an average standard deviation of 7% of the value derived for the temperature, ranging from 2 to 13%. The probability of observing auroral features along the slit is also dependent on the slit orientation, with the slit aligned with the rotational axis producing a higher contrast H_3_^+^ signal, while if the slit aligned with the equator has any H_3_^+^ enhancements rotating along the slit, the observed contrast will be lower.

## 5. Conclusion

This study has revealed that the upper atmosphere of Uranus has cooled consistently between 1992 and 2018, at a rate of 8 ± 1 K yr^−1^. Since this is longer than the 21 year seasonal cycle, it may be linked to auroral Joule heating being modulated by an offset and asymmetric magnetic field, making the northern summer solstice hot and the southern winter solstice cold. However, further observations are required to confirm this hypothesis.

In 2014, during the outbreak of the largest tropospheric storm observed at Uranus in over a decade, the temperatures of the upper atmosphere were observed to be hotter than the previous year. This is suggestive of heating associated with acoustic or gravity waves generated by the turbulent troposphere, as previously observed on Jupiter [15].

The NASA IRTF iSHELL observations of 2016-285 revealed a bright H_3_^+^ intensity enhancement rotating into view on the dawn limb. This feature appears at latitudes consistent with the southern auroral region observed by Voyager 2 [5], and may be auroral in nature. However, since other hypotheses cannot be unambiguously ruled out, the origin of this feature remains ambiguous.

Looking towards the next decade, the James Webb Space Telescope (JWST) promises to transform our understanding of the upper atmosphere of Uranus, its coupling to the magnetic field, and the extent to which it is heated by waves generated in the lower atmosphere. The NIRSPEC instrument [41] on-board the JWST has a two-dimensional field-of-view of 3 × 3^′′^, with each pixel covering 0.1 × 0.1^′′^. This enables us to capture almost the entire disc of Uranus in one exposure, providing unrivalled spatial resolution and sensitivity. As part of the Guaranteed Time Observing programme, a full map of H_3_^+^ emissions across the disc of Uranus will be obtained in the early science phase of the telescope.

Despite 27 years of observations of H_3_^+^ emission from Uranus, our understanding of its upper atmosphere remains in its infancy, and further observations and subsequent modelling are required to form a basic understanding of this enigmatic system. In addition, Uranus is proving a very worthy target for future spacecraft missions.

## Data accessibility

The data are available in public telescope archives, or from H.M. upon request.
